# Poly[[tetra­kis­(μ_2_-pyrazine *N*,*N*′-dioxide-κ^2^ *O*:*O*′)dysprosium(III)] tris­(perchlorate)]

**DOI:** 10.1107/S160053681003182X

**Published:** 2010-08-18

**Authors:** Benjamin G. Quinn-Elmore, James D. Buchner, Keith B. Beach, Jacqueline M. Knaust

**Affiliations:** a Allegheny College, 520 North Main St., Meadville, PA 16335, USA

## Abstract

The title three-dimensional coordination network, {[Dy(C_4_H_4_N_2_O_2_)_4_](ClO_4_)_3_}_*n*_, is isostructural of other lanthanides. The Dy^+3 ^cation lies on a fourfold roto-inversion axis. It is coordinated in a distorted square-anti­prismatic fashion by eight O atoms from bridging pyrazine *N*,*N*′-dioxide ligands. There are two unique pyrazine *N*,*N*′-dioxide ligands. One ring is located around an inversion center, and there is a a twofold rotation axis at the center of the other ring. There are also two unique perchlorate anions. One is centered on a twofold rotation axis and the other on a fourfold roto-inversion axis. The perchlorate anions are located in channels that run perpendicular to (001) and (110) and inter­act with the coordination network through C—H⋯O hydrogen bonds.

## Related literature

For the isostructural La, Ce, Pr, Sm, Eu, Gd, Tb and Y coordination networks, see: Sun *et al.* (2004 ▶). For the isostructural Nd, Ho and Er coordination networks, see: Quinn-Elmore *et al.* (2010 ▶); Buchner *et al.* (2010*a* ▶,*b* ▶), respectively. Detailed background to this study is given in the first article of this series by Quinn-Elmore *et al.* (2010 ▶).
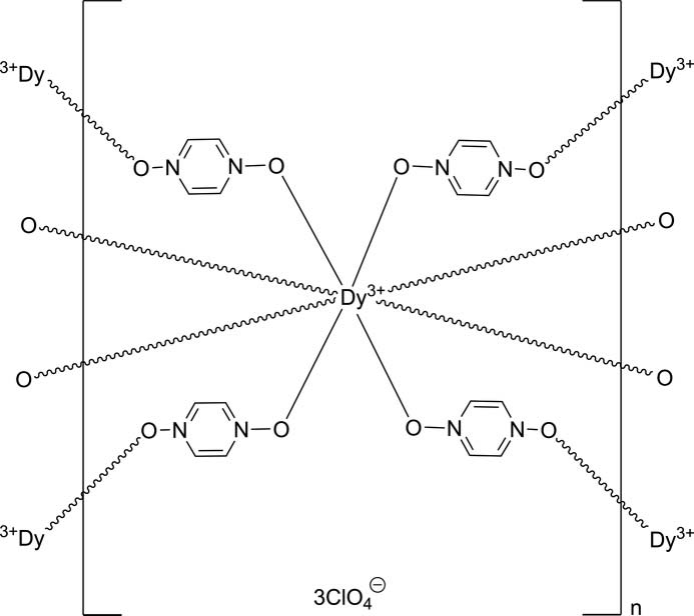


## Experimental

### 

#### Crystal data

[Dy(C_4_H_4_N_2_O_2_)_4_](ClO_4_)_3_
*M* *_r_* = 909.22
Tetragonal, 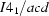

*a* = 15.2553 (4) Å
*c* = 22.6667 (12) Å
*V* = 5275.1 (3) Å^3^
*Z* = 8
Mo *K*α radiation
μ = 3.25 mm^−1^
*T* = 100 K
0.34 × 0.27 × 0.20 mm

#### Data collection

Bruker SMART APEX CCD diffractometer
Absorption correction: multi-scan (*SADABS*; Bruker, 2001 ▶) *T*_min_ = 0.374, *T*_max_ = 0.532
8476 measured reflections
1800 independent reflections
1493 reflections with *I* > 2σ(*I*)
*R*_int_ = 0.018

#### Refinement

*R*[*F* ^2^ > 2σ(*F* ^2^)] = 0.033
*wR*(*F* ^2^) = 0.096
*S* = 0.99
1800 reflections
110 parameters
H-atom parameters constrained
Δρ_max_ = 2.68 e Å^−3^
Δρ_min_ = −1.48 e Å^−3^

### 

Data collection: *SMART* (Bruker, 2007 ▶); cell refinement: *SAINT-Plus* (Bruker, 2007 ▶); data reduction: *SAINT-Plus*; program(s) used to solve structure: *SHELXS97* (Sheldrick, 2008 ▶); program(s) used to refine structure: *SHELXL97* (Sheldrick, 2008 ▶); molecular graphics: *X-SEED* (Barbour, 2001 ▶); software used to prepare material for publication: *X-SEED*.

## Supplementary Material

Crystal structure: contains datablocks I, global. DOI: 10.1107/S160053681003182X/zl2299sup1.cif

Structure factors: contains datablocks I. DOI: 10.1107/S160053681003182X/zl2299Isup2.hkl

Additional supplementary materials:  crystallographic information; 3D view; checkCIF report

## Figures and Tables

**Table 1:** Hydrogen-bond geometry (Å, °)

*D*—H⋯*A*	*D*—H	H⋯*A*	*D*⋯*A*	*D*—H⋯*A*
C2—H2⋯O2^i^	0.95	2.52	3.293 (4)	138
C2—H2⋯O5	0.95	2.40	3.168 (6)	137
C3—H3⋯O1	0.95	2.60	3.329 (4)	134
C3—H3⋯O3	0.95	2.47	3.234 (4)	138
C4—H4⋯O3^ii^	0.95	2.37	3.245 (4)	153

Symmetry codes: (i) 
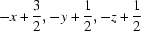
; (ii) 
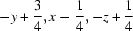
.

**Fig. 1.**
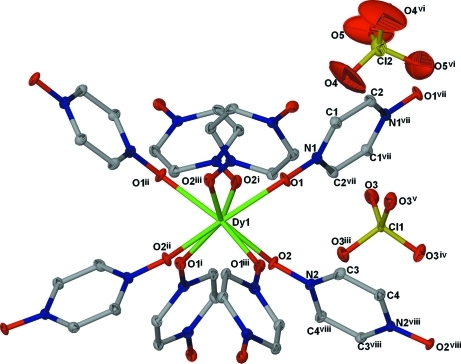
The coordination environment of the Dy+3 cation in title compound with atom labels and 50% probability displacement ellipsoids. Hydrogen atoms have been omitted for clarity. Symmetry codes: (i) y + 1/4, x - 1/4, -z + 3/4; (ii) -y + 3/4, -x + 3/4, -z + 3/4; (iii) -x + 1, -y + 1/2, z; (iv) -y + 3/4, x - 1/4, -z + 1/4; (v) y + 1/4, -x + 3/4, -z + 1/4; (vi) y + 3/4, x - 3/4, -z + 1/4; (vii) -x + 3/2, -y + 1/2, -z + 1/2; (viii) x, -y + 1, -z + 1/2.

## supplementary crystallographic information

### Comment

The description of the structure of the title compound is part of a series of consecutive papers on three-dimensional coordination networks of the type {[Ln(C_4_H_4_N_2_O_2_)_4_](ClO_4_)_3_}_n_, with Ln = Nd (Quinn-Elmore *et al.* 2010), Dy (this publication), Ho (Buchner *et al.* 2010*a*) and Er (Buchner *et al.* 2010*b*), respectively. All four compounds are also isostructural to the previously reported La, Ce, Pr, Sm, Eu, Gd, Tb and Y coordination networks (Sun *et al.* 2004). The background to this study is given in ther first article of this series by Quinn-Elmore *et al.* (2010).

### Experimental

Pyrazine *N*,*N*'-dioxide (0.025 g, 0.223 mmol) was dissolved in deionized water (1.5 ml) and methanol (1.5 ml). An aqueous solution of Dy(ClO_4_)_3_ (0.320 ml of a 0.0868 *M* solution, 0.028 mmol) was diluted with methanol (0.680 ml) and CH_2_Cl_2_ (2.5 ml). The pyrazine *N*,*N*'-dioxide solution was layered over the Dy(ClO_4_)_3_ solution, and the two solutions were allowed to slowly mix. Colorless block-like crystals formed upon the slow evaporation of the resultant solution.

### Refinement

All H atoms were positioned geometrically and refined using a riding model with C—H = 0.95Å and with *U_iso_*(H) = 1.2 times *U*_eq_(C).

### Crystal data

**Table:**

[Dy(C_4_H_4_N_2_O_2_)_4_](ClO_4_)_3_	*D*_x_ = 2.290 Mg m^−^^3^
*M**_r_* = 909.22	Mo *K*α radiation, λ = 0.71073 Å
Tetragonal, *I*4_1_/*a**c**d*	Cell parameters from 4338 reflections
Hall symbol: -I 4bd 2c	θ = 2.7–30.5°
*a* = 15.2553 (4) Å	µ = 3.25 mm^−^^1^
*c* = 22.6667 (12) Å	*T* = 100 K
*V* = 5275.1 (3) Å^3^	Block, colourless
*Z* = 8	0.34 × 0.27 × 0.20 mm
*F*(000) = 3560	

### Data collection

**Table:**

Bruker SMART APEX CCD diffractometer	1800 independent reflections
Radiation source: fine-focus sealed tube	1493 reflections with *I* > 2σ(*I*)
graphite	*R*_int_ = 0.018
ω scans	θ_max_ = 30.5°, θ_min_ = 2.6°
Absorption correction: multi-scan (*SADABS*; Bruker, 2001)	*h* = −21→19
*T*_min_ = 0.374, *T*_max_ = 0.532	*k* = −3→21
8476 measured reflections	*l* = −20→25

### Refinement

**Table:**

Refinement on *F*^2^	Primary atom site location: structure-invariant direct methods
Least-squares matrix: full	Secondary atom site location: difference Fourier map
*R*[*F*^2^ > 2σ(*F*^2^)] = 0.033	Hydrogen site location: inferred from neighbouring sites
*wR*(*F*^2^) = 0.096	H-atom parameters constrained
*S* = 0.99	*w* = 1/[σ^2^(*F*_o_^2^) + (0.0554*P*)^2^ + 37.1849*P*] where *P* = (*F*_o_^2^ + 2*F*_c_^2^)/3
1800 reflections	(Δ/σ)_max_ < 0.001
110 parameters	Δρ_max_ = 2.68 e Å^−^^3^
0 restraints	Δρ_min_ = −1.48 e Å^−^^3^

### Special details

**Table:**

Geometry. All e.s.d.'s (except the e.s.d. in the dihedral angle between two l.s. planes) are estimated using the full covariance matrix. The cell e.s.d.'s are taken into account individually in the estimation of e.s.d.'s in distances, angles and torsion angles; correlations between e.s.d.'s in cell parameters are only used when they are defined by crystal symmetry. An approximate (isotropic) treatment of cell e.s.d.'s is used for estimating e.s.d.'s involving l.s. planes.
Refinement. Refinement of *F*^2^ against ALL reflections. The weighted *R*-factor *wR* and goodness of fit *S* are based on *F*^2^, conventional *R*-factors *R* are based on *F*, with *F* set to zero for negative *F*^2^. The threshold expression of *F*^2^ > σ(*F*^2^) is used only for calculating *R*-factors(gt) *etc*. and is not relevant to the choice of reflections for refinement. *R*-factors based on *F*^2^ are statistically about twice as large as those based on *F*, and *R*- factors based on ALL data will be even larger.

### Fractional atomic coordinates and isotropic or equivalent isotropic displacement parameters (Å^2^)

**Table:**

	*x*	*y*	*z*	*U*_iso_*/*U*_eq_	
Dy1	0.5000	0.2500	0.3750	0.00560 (12)	
Cl1	0.5000	0.2500	0.1250	0.0113 (4)	
Cl2	0.72654 (6)	−0.02346 (6)	0.1250	0.0281 (3)	
O1	0.59121 (12)	0.21876 (14)	0.29510 (10)	0.0155 (4)	
O2	0.53217 (14)	0.39451 (12)	0.34383 (10)	0.0153 (4)	
O3	0.57642 (16)	0.24419 (16)	0.16207 (12)	0.0251 (6)	
O4	0.6466 (4)	−0.0162 (4)	0.1506 (4)	0.137 (3)	
O5	0.7912 (4)	−0.0056 (4)	0.1658 (4)	0.129 (3)	
N1	0.66943 (16)	0.23424 (16)	0.27415 (13)	0.0143 (5)	
N2	0.52711 (16)	0.44491 (15)	0.29779 (12)	0.0126 (5)	
C1	0.70896 (18)	0.17248 (18)	0.24020 (14)	0.0151 (6)	
H1	0.6807	0.1179	0.2335	0.018*	
C2	0.78920 (18)	0.18838 (18)	0.21566 (14)	0.0150 (6)	
H2	0.8161	0.1453	0.1914	0.018*	
C3	0.5260 (2)	0.41183 (17)	0.24253 (15)	0.0158 (6)	
H3	0.5252	0.3501	0.2368	0.019*	
C4	0.52588 (19)	0.46669 (17)	0.19470 (15)	0.0152 (6)	
H4	0.5250	0.4428	0.1560	0.018*	

### Atomic displacement parameters (Å^2^)

**Table:**

	*U*^11^	*U*^22^	*U*^33^	*U*^12^	*U*^13^	*U*^23^
Dy1	0.00587 (12)	0.00587 (12)	0.0051 (2)	−0.00030 (6)	0.000	0.000
Cl1	0.0138 (5)	0.0138 (5)	0.0061 (11)	0.000	0.000	0.000
Cl2	0.0263 (4)	0.0263 (4)	0.0316 (9)	−0.0106 (5)	0.0033 (3)	−0.0033 (3)
O1	0.0097 (8)	0.0235 (10)	0.0132 (12)	−0.0029 (7)	0.0045 (7)	−0.0042 (8)
O2	0.0247 (10)	0.0113 (8)	0.0100 (13)	−0.0020 (7)	−0.0023 (8)	0.0048 (7)
O3	0.0179 (11)	0.0433 (15)	0.0142 (15)	0.0047 (9)	−0.0040 (10)	−0.0023 (9)
O4	0.066 (3)	0.114 (5)	0.230 (7)	−0.010 (3)	0.097 (5)	−0.016 (5)
O5	0.076 (4)	0.099 (4)	0.212 (9)	0.003 (3)	−0.058 (5)	−0.076 (5)
N1	0.0113 (10)	0.0182 (10)	0.0134 (16)	−0.0011 (8)	0.0010 (9)	0.0001 (10)
N2	0.0149 (10)	0.0121 (10)	0.0108 (15)	−0.0003 (8)	−0.0013 (9)	0.0018 (9)
C1	0.0154 (12)	0.0159 (12)	0.0142 (17)	−0.0005 (9)	0.0008 (10)	−0.0029 (10)
C2	0.0137 (12)	0.0182 (13)	0.0130 (17)	−0.0004 (9)	0.0006 (10)	−0.0040 (11)
C3	0.0193 (13)	0.0110 (11)	0.0169 (18)	−0.0022 (10)	−0.0026 (12)	0.0005 (10)
C4	0.0205 (13)	0.0116 (11)	0.0135 (17)	−0.0001 (10)	0.0006 (11)	0.0000 (10)

### Geometric parameters (Å, °)

**Table:**

Dy1—O1^i^	2.333 (2)	Cl2—O5^vi^	1.380 (6)
Dy1—O1^ii^	2.333 (2)	O1—N1	1.306 (3)
Dy1—O1	2.333 (2)	O2—N2	1.298 (3)
Dy1—O1^iii^	2.333 (2)	N1—C1	1.358 (4)
Dy1—O2^i^	2.3665 (19)	N1—C2^vii^	1.358 (4)
Dy1—O2^ii^	2.3665 (19)	N2—C3	1.351 (4)
Dy1—O2	2.3665 (19)	N2—C4^viii^	1.359 (3)
Dy1—O2^iii^	2.3665 (19)	C1—C2	1.366 (4)
Cl1—O3	1.440 (2)	C1—H1	0.9500
Cl1—O3^iv^	1.440 (2)	C2—N1^vii^	1.358 (4)
Cl1—O3^iii^	1.440 (2)	C2—H2	0.9500
Cl1—O3^v^	1.440 (2)	C3—C4	1.370 (4)
Cl2—O4^vi^	1.355 (4)	C3—H3	0.9500
Cl2—O4	1.355 (4)	C4—N2^viii^	1.359 (3)
Cl2—O5	1.380 (6)	C4—H4	0.9500
			
O1^i^—Dy1—O1^ii^	78.16 (11)	O3^iv^—Cl1—O3^iii^	109.92 (11)
O1^i^—Dy1—O1	147.81 (10)	O3—Cl1—O3^v^	109.92 (11)
O1^ii^—Dy1—O1	111.03 (11)	O3^iv^—Cl1—O3^v^	108.6 (2)
O1^i^—Dy1—O1^iii^	111.03 (11)	O3^iii^—Cl1—O3^v^	109.91 (11)
O1^ii^—Dy1—O1^iii^	147.81 (10)	O4^vi^—Cl2—O4	109.3 (6)
O1—Dy1—O1^iii^	78.16 (11)	O4^vi^—Cl2—O5	114.0 (4)
O1^i^—Dy1—O2^i^	80.49 (7)	O4—Cl2—O5	109.9 (5)
O1^ii^—Dy1—O2^i^	72.64 (7)	O4^vi^—Cl2—O5^vi^	109.9 (5)
O1—Dy1—O2^i^	73.66 (7)	O4—Cl2—O5^vi^	114.0 (4)
O1^iii^—Dy1—O2^i^	138.16 (7)	O5—Cl2—O5^vi^	99.7 (8)
O1^i^—Dy1—O2^ii^	72.64 (7)	N1—O1—Dy1	142.21 (18)
O1^ii^—Dy1—O2^ii^	80.49 (7)	N2—O2—Dy1	141.19 (17)
O1—Dy1—O2^ii^	138.16 (7)	O1—N1—C1	119.1 (2)
O1^iii^—Dy1—O2^ii^	73.66 (7)	O1—N1—C2^vii^	121.3 (2)
O2^i^—Dy1—O2^ii^	145.25 (11)	C1—N1—C2^vii^	119.5 (2)
O1^i^—Dy1—O2	73.66 (7)	O2—N2—C3	121.7 (2)
O1^ii^—Dy1—O2	138.16 (7)	O2—N2—C4^viii^	119.2 (3)
O1—Dy1—O2	80.49 (7)	C3—N2—C4^viii^	119.1 (3)
O1^iii^—Dy1—O2	72.64 (7)	N1—C1—C2	120.4 (3)
O2^i^—Dy1—O2	72.71 (10)	N1—C1—H1	119.8
O2^ii^—Dy1—O2	118.40 (10)	C2—C1—H1	119.8
O1^i^—Dy1—O2^iii^	138.16 (7)	N1^vii^—C2—C1	120.1 (3)
O1^ii^—Dy1—O2^iii^	73.66 (7)	N1^vii^—C2—H2	120.0
O1—Dy1—O2^iii^	72.64 (7)	C1—C2—H2	120.0
O1^iii^—Dy1—O2^iii^	80.49 (7)	N2—C3—C4	120.4 (2)
O2^i^—Dy1—O2^iii^	118.40 (10)	N2—C3—H3	119.8
O2^ii^—Dy1—O2^iii^	72.71 (10)	C4—C3—H3	119.8
O2—Dy1—O2^iii^	145.25 (11)	N2^viii^—C4—C3	120.5 (3)
O3—Cl1—O3^iv^	109.91 (11)	N2^viii^—C4—H4	119.8
O3—Cl1—O3^iii^	108.6 (2)	C3—C4—H4	119.8

Symmetry codes: (i) *y*+1/4, *x*−1/4, −*z*+3/4; (ii) −*y*+3/4, −*x*+3/4, −*z*+3/4; (iii) −*x*+1, −*y*+1/2, *z*; (iv) −*y*+3/4, *x*−1/4, −*z*+1/4; (v) *y*+1/4, −*x*+3/4, −*z*+1/4; (vi) *y*+3/4, *x*−3/4, −*z*+1/4; (vii) −*x*+3/2, −*y*+1/2, −*z*+1/2; (viii) *x*, −*y*+1, −*z*+1/2.

### Hydrogen-bond geometry (Å, °)

**Table:**

*D*—H···*A*	*D*—H	H···*A*	*D*···*A*	*D*—H···*A*
C2—H2···O2^vii^	0.95	2.52	3.293 (4)	138.
C2—H2···O5	0.95	2.40	3.168 (6)	137.
C3—H3···O1	0.95	2.60	3.329 (4)	134.
C3—H3···O3	0.95	2.47	3.234 (4)	138.
C4—H4···O3^iv^	0.95	2.37	3.245 (4)	153.

Symmetry codes: (vii) −*x*+3/2, −*y*+1/2, −*z*+1/2; (iv) −*y*+3/4, *x*−1/4, −*z*+1/4.
